# Speech-Language Pathology-Related Disorders in Adults with Brain Tumours: A Narrative Review

**DOI:** 10.3390/biomedicines14081768

**Published:** 2026-08-06

**Authors:** Sara Nordio, Pier Franco Conte

**Affiliations:** IRCCS San Camillo Hospital, 30126 Venice, Italy

**Keywords:** brain tumours, speech and language pathology, aphasia, dysarthria, dysphagia

## Abstract

Brain tumours frequently involve eloquent cortical and subcortical networks, leading to speech-language pathology (SLP) disorders, including aphasia, dysarthria, and dysphagia. Evidence remains fragmented and largely derived from stroke-based frameworks that may not fully reflect neuro-oncological populations. This review synthesised literature on SLP disorders in adults with primary brain tumours across the pre-operative, peri-operative, and post-operative phases. Studies were identified through searches of PubMed/MEDLINE, Embase, CINAHL, Cochrane Library, and Web of Science (2000–2026) and narratively synthesised by domain and clinical contexts. Aphasia is the most studied domain and is typically mild and anomic, particularly in low-grade gliomas. Dysarthria commonly arises from damage to the ventral premotor cortex, periventricular white matter, and corticobulbar fibres, with articulatory severity correlating with subcortical tract involvement. Although less frequently investigated, dysphagia emerges as a clinically relevant complication, especially in posterior fossa and brainstem tumours, where upper oesophageal sphincter dysfunction and aspiration risk may occur. Intra-operative mapping improves functional outcomes, but tumour-specific assessment tools are lacking across all domains. SLP disorders in patients with brain tumours differ substantially from those observed after stroke and have significant consequences for communication, swallowing, and quality of life. There is a critical need for standardised, neuro-oncology-specific assessment and rehabilitation frameworks.

## 1. Introduction

Primary brain tumours affect approximately seven to eight per 100,000 individuals annually in high-income countries, with gliomas accounting for nearly 30% of all central nervous system tumours and 80% of malignant cases [1]. Although advances in neurosurgical techniques, particularly awake craniotomy and intra-operative electrical stimulation mapping, have improved both survival rates and the extent of resection, they have simultaneously highlighted the acute and long-term functional consequences of surgery on language, speech, and swallowing systems [2,3].

Speech-language pathology (SLP) disorders in patients with brain tumours manifest across three principal domains: (1) aphasia, broadly defined as an acquired impairment of language processing [4]; (2) dysarthria, referring to motor speech disorders arising from neuromotor dysfunction [5]; and (3) dysphagia, a swallowing disorder reflecting dysfunction within complex, integrated sensorimotor and cortico-subcortical coordination networks [6]. Dysphagia is of particular clinical concern due to its strong association with malnutrition, dehydration, aspiration pneumonia, prolonged hospitalisation, reduced quality of life, and increased mortality across neurological populations [7,8]. These disorders may occur in isolation or in combination; moreover, they may pre-date surgery due to tumour-induced mass effect or infiltration, or be exacerbated or newly induced by surgical resection, radiotherapy, and corticosteroid tapering [9].

Unlike acute cerebrovascular accidents (stroke), brain tumours often evolve gradually, permitting partial neuroplastic reorganisation and functional redistribution [10]. This fundamental pathophysiological difference leads to distinct clinical phenotypes. Post-operative aphasia following tumour resection is typically milder, predominantly presenting with anomic profiles and relatively preserved comprehension compared to vascular aphasia syndromes [11]. Similarly, motor speech and swallowing impairments are influenced not only by focal tissue destruction but also by tract displacement, peritumoural infiltration, and dynamic network reorganisation [3]. Despite a growing body of research on language, speech, and swallowing outcomes in neuro-oncology, available evidence remains fragmented across disciplines and clinical domains.

Beyond direct neurological deficits, speech-language and swallowing disorders substantially impair health-related quality of life (HRQoL) in adults with primary brain tumours. Communication deficits compromise social participation, employment, interpersonal relationships, personal autonomy, and emotional well-being, while dysphagia negatively affects nutritional status, mealtime participation, and the perceived dignity and safety of eating [12]. Patients with gliomas frequently report reduced quality of life across cognitive, emotional, physical, and social domains, with communication difficulties acting as a major driver of functional disability and psychosocial burden [12,13]. Notably, even subtle language impairments, such as word-finding delays or discourse inefficiencies, can hinder a return to work, complex decision-making, and the preservation of social identity, despite otherwise preserved global neurological functioning [14]. Similarly, dysphagia can lead to social withdrawal, mealtime anxiety, reduced oral intake, and heightened caregiver burden, thereby further undermining the overall quality of life [12]. As survival rates improve and neuro-oncological care increasingly prioritises functional preservation, safeguarding communication and swallowing abilities has become a cornerstone of patient-centred rehabilitation and long-term survivorship care.

Despite the clinical relevance and frequency of these disorders, the current evidence base remains fragmented, hindering the development of integrated rehabilitation pathways and fostering inconsistent clinical practice. Although systematic reviews have separately addressed post-neurosurgical language outcomes [9], and specific cohorts have examined dysphagia in neuro-oncological rehabilitation settings [15], no comprehensive review has simultaneously evaluated all three SLP domains—aphasia, dysarthria, and dysphagia—across the complete disease trajectory within a unified synthesis. This underscores the need for an integrated overview of current knowledge regarding their clinical characteristics, assessment, and rehabilitative management.

The specific objectives of this narrative review were to (1) characterise the prevalence, phenotype, and severity of dysphagia, dysarthria, and aphasia in adults with primary brain tumours, (2) delineate their underlying neuroanatomical and pathophysiological correlations, (3) summarise the current assessment tools and rehabilitative intervention approaches, and (4) identify critical evidence gaps to guide future research priorities.

## 2. Literature Search Strategy

A narrative review methodology was adopted to enable the integration and critical synthesis of heterogeneous evidence across observational cohort studies, interventional trials, and intra-operative mapping series. This review was conducted and reported in accordance with established methodological recommendations for narrative literature reviews described by Ferrari [16] and Sukhera [17].

### 2.1. Search Strategy and Databases

A comprehensive, structured literature search was performed across five electronic databases: PubMed/MEDLINE, Embase, CINAHL, Cochrane Library, and Web of Science. The search covered literature published from 1 January 2000 to 31 March 2026. To identify all relevant studies, a structured and comprehensive search query was executed in PubMed. The search strategy was constructed using a combination of Medical Subject Headings (MeSH terms) to capture indexed literature and free-text keywords restricted to the title/abstract ([Title/Abstract]) field to ensure sensitivity and specificity toward the primary scope of the review. Truncation symbols (*) were applied to capture terminology variants (e.g., American vs. British spelling for “tumor/tumour”).

The final search architecture combined three core conceptual blocks using the Boolean operator AND:

1. Target Pathology and Oncological Domains: MeSH terms and title/abstract keywords capturing primary brain tumours and high-grade lesions (“Brain Neoplasms”[MeSH] OR “Glioma”[MeSH] OR “Glioblastoma”[MeSH] OR “Brain Tumour*”[Title/Abstract] OR “Brain Tumor*”[Title/Abstract]).
2. Functional and Logopedic Domains: Terms encompassing speech, language, and swallowing impairment (“Speech-Language Pathology”[MeSH] OR “Language Disorders”[MeSH] OR “Aphasia”[MeSH] OR “Dysarthria”[MeSH] OR “Deglutition Disorders”[MeSH] OR “Dysphagia”[Title/Abstract] OR “Anomia”[Title/Abstract]).
3. Clinical Management and Intervention Contexts: Terms targeting diagnostic evaluation, therapeutic protocols, and surgical/rehabilitative pathways (“Assessment”[Title/Abstract] OR “Rehabilitation”[MeSH] OR “Therapy”[Title/Abstract] OR “Awake Mapping”[Title/Abstract]).

The complete search syntax was formatted as follows:

(“Brain Neoplasms”[MeSH] OR “Glioma”[MeSH] OR “Glioblastoma”[MeSH] OR “Brain Tumour*”[Title/Abstract] OR “Brain Tumor*”[Title/Abstract]) AND (“Speech-Language Pathology”[MeSH] OR “Language Disorders”[MeSH] OR “Aphasia”[MeSH] OR “Dysarthria”[MeSH] OR “Deglutition Disorders”[MeSH] OR “Dysphagia”[Title/Abstract] OR “Anomia”[Title/Abstract]) AND (“Assessment”[Title/Abstract] OR “Rehabilitation”[MeSH] OR “Therapy”[Title/Abstract] OR “Awake Mapping”[Title/Abstract]).

Full, unmitigated database-specific search strings (including syntax variations adapted for EMBASE and Web of Science) were executed and cross-referenced. Additional manual searches were performed by screening the reference lists of key reviews and eligible primary articles.

### 2.2. Eligibility and Selection Criteria

Eligible studies included the following: (1) adult patients (≥18 years) with primary brain tumours (e.g., low-grade and high-grade gliomas, meningiomas); (2) reporting functional, clinical, or intra-operative outcomes related to aphasia, dysarthria, or dysphagia; and (3) published in peer-reviewed English-language journals.

Studies involving metastatic brain disease exclusively, paediatric cohorts, non-human models, or non-peer-reviewed commentaries were excluded. Data were synthesised thematically according to the SLP domain, tumour location, assessment tools, and clinical outcomes. Due to substantial clinical and methodological heterogeneity, findings are presented as a structured narrative synthesis supported by descriptive aggregation of the reported frequencies.

## 3. Results

The literature search and screening process yielded a final selection of studies investigating speech, language, and swallowing disorders in patients with primary brain tumours. Initial database querying identified a total of 80 records; following duplicate removal and rigorous title, abstract, and full-text screening against the predefined inclusion criteria, 16 studies were ultimately included in this review. A comprehensive overview of the design, sample sizes, patient populations, clinical contexts, and target SLP domains of all included studies is summarised in Table 1.

As shown in Table 1, studies on SLP disorders in neuro-oncology have predominantly focused on patients with gliomas involving language-eloquent regions of the left hemisphere, including in the setting of awake craniotomy and intra-operative language mapping procedures [2,3,18,23,25]. Across the included literature, observational cohort studies and intra-operative mapping series from tertiary neurosurgical centres predominated. While a substantial proportion of pioneering awake surgery and language mapping series originated from European institutions, significant research contributions and clinical protocols are also actively conducted across North American centres. However, a notable gap remains in the formal tracking of ongoing interventional protocols via registries such as ClinicalTrials.gov, as the current literature predominantly reflects published observational data.

Overall, the primary SLP domains investigated across the literature were unevenly distributed:

- Aphasia and language network dysfunction represented the most extensively investigated domain, particularly in the context of awake craniotomy, intra-operative direct electrical stimulation, and post-operative neuroplasticity [2,3,18,23,25,28].
- Dysarthria was substantially less frequently addressed as an isolated primary outcome, being most commonly described within broader motor speech or combined neurological deficits [21].
- Dysphagia emerged as the least explored domain, with available evidence mainly clustered around posterior fossa/brainstem lesions, post-operative surgical complications, and specialised inpatient neuro-rehabilitation settings [15].

A comparative synthesis of the shared neuroanatomical substrates, cortical/subcortical pathways, and clinical functional profiles across aphasia, dysarthria, and dysphagia is provided in Table 2. The specific findings for each SLP domain are detailed in the following subsections.

### 3.1. Aphasia in Brain Tumours

#### 3.1.1. Pre-Operative Language Impairment

Pre-operative language impairment is reported in 12–40% of patients with left-hemisphere gliomas across the included studies [9]. This variability in prevalence reflects differences in assessment tools, impairment thresholds, and tumour location within distributed language networks.

Prospective studies in low-grade glioma cohorts suggest that subtle lexical and executive-linguistic deficits may represent the earliest manifestations of tumour-related language dysfunction; specifically, impairments in confrontation naming and verbal fluency are frequently observed despite largely preserved global language scores.

Moreover, a pre-operative language status carries significant prognostic relevance. Both baseline language impairment and intra-operative speech disturbances are associated with early post-operative deficits, highlighting their predictive value in surgical planning [24]. Similarly, Fang et al. [24] proposed a tumour location-based classification of language risk, stratifying gliomas according to their proximity to cortical and subcortical language networks, which carries direct implications for expected post-operative outcomes.

Indeed, anomia may represent an early and sometimes isolated sign of tumour-related language dysfunction, even in lesions located outside classical language areas [28].

#### 3.1.2. Post-Operative Aphasia: Type and Severity

Consistently across studies, anomic aphasia was the most frequent post-operative subtype (approximately 47–49%), while global aphasia remained rare (3–5%), markedly lower than rates reported in vascular stroke populations [11]. Comparative evidence highlights clear differences between tumour-related and acute neurological aphasia profiles: in contrast to stroke cohorts, where global and Broca’s aphasia predominate [21], tumour populations are characterised by milder, more selective deficits. Klebic et al. further noted that intracerebral tumours produce more severe language impairment than extracerebral lesions, with high-grade gliomas being associated with more pronounced semantic dysfunction [28].

Deficit severity was predominantly mild, with most patients showing mild-range aphasia quotients on the Western Aphasia Battery (WAB; Kertesz et al., 2006) [29], and fewer cases presenting with moderate or severe deficits. Davie et al. (2009) [11] reported no strong association between tumour grade or surgical variables and aphasia severity, whereas Ilmberger et al. (2008) [19] identified pre-operative aphasia and subcortical tract involvement as key predictors of post-operative impairment. Importantly, more sensitive early post-operative assessments reveal a substantially higher burden of transient language dysfunction. In fact, up to 73% of patients exhibit language impairment within 24 h post-surgery, predominantly affecting lexical retrieval and fluency, with most recovering within three months [26]. Moreover, patients with presumed LGG showed increased speech disfluencies after surgery, most notably a higher frequency of fillers in spontaneous speech [26]. The results from this pivotal study, reference [26], demonstrated a post-operative decline in word fluency performance, an increase in the proportion of participants classified with lexical retrieval impairment, and a greater number of individuals reporting subjective changes in language and communication. Those with lexical retrieval impairment showed a higher proportion of false starts compared to their unimpaired peers, while differences in temporal speech measures (such as articulation and speech rate) favoured participants without reported language changes. These findings, derived from spontaneous speech measures, are further complemented by evidence from structured confrontation naming tasks and detailed error analyses, which provide a more fine-grained characterisation of underlying linguistic mechanisms. Naming error analysis in 87 patients undergoing awake brain tumour surgery showed that errors during naming tasks reliably reflect underlying semantic and phonological impairments, as their frequency correlated with domain-specific neuropsychological test performance [27]. At the group level, post-surgical outcomes indicated an overall decline in language functions; however, individual-level analyses revealed that more patients either improved or remained stable than worsened. Additionally, patients with LGG demonstrated more favourable cognitive outcomes after surgery compared to those with HGG, a difference likely related to greater neuroplasticity capacity [27].

#### 3.1.3. Subcortical Language Networks

Intra-operative stimulation studies have consistently identified five principal subcortical language pathways: the arcuate fasciculus supports phonological processing (with stimulation inducing phonemic paraphasia), the inferior fronto-occipital fasciculus is primarily associated with semantic processing (eliciting semantic paraphasia when disrupted), and the subcallosal fasciculus contributes to speech initiation and executive-linguistic control [2,3]. Additional pathways include the frontoparietal phonological loop, involved in verbal working memory and articulatory planning, and ventral premotor/periventricular fibres, critical for motor speech execution. Disruption of these networks produces distinct and reproducible language deficits, providing a functional atlas for intra-operative decision-making [2,18].

#### 3.1.4. Assessment Approaches for Aphasia

The most commonly used language assessment tools include the WAB [29], the Boston Diagnostic Aphasia Examination (BDAE) [30], and the Boston Naming Test [31]. However, all were derived from stroke populations and consequently lack sensitivity to subtle, network-level impairments that are typical of gliomas. Several studies have highlighted that patients with LGG may perform within normal limits on conventional aphasia batteries while exhibiting marked deficits on high-level language tasks, including metaphor comprehension and verbal abstraction [22]. Klebic et al. [28] further emphasised the importance of pragmatic and feasibility considerations in clinical settings, noting that short-form batteries are often utilised pre-operatively, although their sensitivity to subtle deficits remains limited.

Overall, the literature indicates a shift towards network-sensitive and high-level language assessments, although no tool has yet been specifically validated for brain tumour populations.

#### 3.1.5. Clinical Implications and Recovery

Recovery from aphasia after tumour resection is generally favourable, particularly in patients with LGG. Duffau et al. [3] reported near-complete recovery at three months in the majority of patients, reflecting a combination of oedema resolution, diaschisis reversal, and long-term neuroplastic reorganisation involving perilesional and contralateral networks. Recovery is strongly influenced by subcortical connectivity: the integrity of dorsal stream pathways, particularly the superior longitudinal fasciculus and arcuate fasciculus, has been identified as a critical determinant of language outcomes and recovery following glioma surgery [32]. A significant relationship has been observed between lesion proximity to these tracts and recovery time, with tumour-to-arcuate fasciculus distances below 3 mm associated with persistent post-operative impairment [24]. Furthermore, tumour topology plays a crucial role. Lesions involving posterior dorsal language pathways are associated with poorer recovery and more persistent deficits, suggesting limited compensatory capacity within these regions. Conversely, anterior and inferior language networks appear to demonstrate greater functional plasticity and reorganisation potential [33].

In high-grade gliomas, recovery is generally less complete and persistent deficits are more frequent, particularly when resection margins involve critical subcortical pathways. Adjuvant oncological treatments, including radiotherapy, may further contribute to long-term cognitive-linguistic decline [34].

### 3.2. Dysarthria in Brain Tumours

#### 3.2.1. Epidemiology and Neuroanatomical Correlates

Dysarthria is reported in 20 to 65% of patients in the immediate post-operative period, with variability primarily driven by tumour location, extent of resection, and involvement of corticobulbar pathways [25]. The highest incidence is observed in tumours affecting the left inferior frontal gyrus, primary motor cortex, ventral premotor cortex, and periventricular white matter [25]. In awake mapping studies, stimulation of ventral premotor and periventricular fibres reliably induces dysarthria or anarthria, confirming their critical role in motor speech execution [2,3].

Post-operative dysarthria in glioma patients is most frequently described as a mixed spastic–flaccid phenotype, reflecting combined disruption of the upper and lower corticobulbar pathways. Conversely, ataxic features are less frequently reported and typically stem from posterior fossa involvement, whereas hypokinetic characteristics may emerge in basal ganglia tumours [25].

Although data specific to brain tumour populations remain limited, comparative neurological studies suggest a consistent relationship between lesion site and dysarthria subtype, reinforcing the role of anatomical networks in shaping motor speech profiles [21]. In neuro-oncology, these patterns appear to follow a similar framework, with tumour location serving as the primary determinant of the dysarthria phenotype.

A well-described post-operative syndrome affecting motor speech is supplementary motor area (SMA) syndrome. This condition is characterised by transient speech reduction, initiation deficits, and mild dysarthria or transcortical motor aphasia, most commonly following superior frontal resections. Across surgical series, including Duffau et al. [3], SMA syndrome was consistently observed following resections involving the medial frontal cortex. The syndrome typically resolves within three months, supporting a functional rather than structural mechanism of impairment.

Recovery is thought to reflect reorganisation within medial frontal and cingulate networks, alongside compensatory recruitment of contralateral SMA regions, highlighting the remarkable plasticity of medial motor speech systems.

#### 3.2.2. Assessment Approaches for Dysarthria

Assessment of dysarthria in brain tumour populations remains limited and largely non-standardised. Only a minority of studies have employed validated tools such as the Frenchay Dysarthria Assessment [35] or structured perceptual intelligibility scales.

#### 3.2.3. Clinical Implications and Recovery

To date, no studies have systematically investigated rehabilitative interventions for dysarthria specifically in patients with brain tumours.

### 3.3. Dysphagia in Brain Tumours

#### 3.3.1. Epidemiology and Clinical Presentation

Dysphagia is the least investigated SLP domain; reported prevalence ranges from 15% to 48%, with higher rates observed in patients with infratentorial tumours (posterior fossa, brainstem, cerebellopontine angle), large supratentorial lesions with mass effect, and bilateral cortical involvement [15]. Across studies, the most frequently described phenotype is oropharyngeal dysphagia, characterised by pharyngeal residue, reduced airway protection, impaired hyolaryngeal excursion, and upper oesophageal sphincter (UES) dysfunction [15,21].

Lesion location serves as the primary determinant of severity. Brainstem involvement, particularly affecting medullary structures, is consistently associated with more severe swallowing impairment and a higher aspiration risk [15]. In these cases, the clinical presentation closely resembles brainstem stroke dysphagia, featuring impaired pharyngeal clearance and reduced laryngeal elevation; however, no significant difference in swallowing function has been observed between patients with benign versus malignant brain tumours [36]. Cognitive impairment, tumour-related neurological deficits, and treatment side effects (radiotherapy-induced fibrosis, oedema, and xerostomia) are frequently cited as additional contributors to swallowing dysfunction, underscoring the multifactorial nature of dysphagia in neuro-oncology [37].

#### 3.3.2. Neuroanatomical Correlates and Mechanisms

Swallowing impairment in brain tumour patients reflects the disruption of a distributed cortico-subcortical network involving both cortical control systems and brainstem central pattern generators. A critical mechanism identified across studies is cricopharyngeal dysfunction (CPD), particularly in lesions involving the medulla or exerting mass effect on posterior fossa structures. Disruption of the nucleus tractus solitarius and dorsal motor nucleus of the vagus leads to impaired coordination of UES relaxation and reduced bolus passage, resulting in post-swallow residue and a heightened aspiration risk [37]. Moreover, cortical and subcortical lesions may additionally contribute through impaired sensorimotor integration, reduced oral bolus control, and delayed swallow initiation [6]. Evidence from mixed neurological populations supports a strong association between dysphagia and dysarthria, reflecting shared involvement of corticobulbar pathways. Jani et al. [21] reported high rates of co-occurrence, particularly in conditions affecting bulbar motor systems; in contrast, cerebellar involvement was less consistently associated with swallowing impairment despite the frequent presence of dysarthria.

#### 3.3.3. Assessment Approaches for Dysphagia

Instrumental assessment was performed in the majority of studies, with a videofluoroscopic swallowing study (VFSS) and fibreoptic endoscopic evaluation of swallowing (FEES) being the most frequently used techniques. The Penetration-Aspiration Scale (PAS) [38] was the most commonly reported outcome measure, with scores typically ranging from moderate to severe impairment in symptomatic patients. Bedside clinical swallowing evaluation (CSE) [39] was frequently used as an initial screening tool.

#### 3.3.4. Clinical Implications and Recovery

From a rehabilitation perspective, SLP-led interventions were variably described and included compensatory strategies (e.g., postural adjustments and diet modification), swallowing manoeuvres such as the effortful swallow and the Mendelsohn manoeuvre [40] and, in selected cases, targeted interventions for CPD, including the chin tuck against resistance and the Shaker manoeuvre [41]. Enteral feeding via Percutaneous Endoscopic Gastrostomy (PEG) or Radiologically Inserted Gastrostomy (RIG) was reported in severe or persistent cases, particularly those involving the involvement of or during intensive oncological treatment [37].

Preliminary evidence from inpatient rehabilitation settings suggests that dysphagia in brain tumour patients is responsive to structured intervention, with patients demonstrating functional swallowing gains comparable to those achieved by stroke patients undergoing rehabilitation [15].

## 4. Discussion

### 4.1. Shared Network Mechanisms Across SLP Disorders and Assessment Challenges in Neuro-Oncology

This narrative review demonstrates that SLP disorders in primary brain tumours are best conceptualised as emergent manifestations of distributed connectome disruption rather than discrete focal deficits. Aphasia, dysarthria, and dysphagia reflect partially overlapping cortico-subcortical systems subserving language, motor speech, and swallowing, with differential vulnerability to tumour infiltration, surgical manipulation, and adjuvant therapies. However, a critical synthesis of the literature reveals a persistent fragmentation: only a limited number of publications simultaneously address communication and swallowing disorders across the entire oncological trajectory [9,25].

This siloed approach obscures the cumulative functional burden on patients and hampers the development of integrated rehabilitation pathways. From a neurobiological perspective, tumour-related SLP disorders diverge fundamentally from post-stroke vascular insults. While acute vascular occlusion causes abrupt focal tissue necrosis, following specific arterial territories (e.g., MCA), slow-growing primary brain tumours (particularly lower-grade gliomas) induce progressive, non-linear network reconfiguration that is characterised by neuroplastic adaptation, functional redistribution, and transient diaschisis [2,3,10]. Conversely, high-grade gliomas exert rapid mass effects, vasogenic oedema, and local infiltration, disrupting white matter tracts before functional reorganisation can take place. These distinct pathophysiological cascades lead to radically different clinical presentations, recovery dynamics, and assessment vulnerabilities between stroke and neuro-oncology populations. Specifically, key pathophysiological and clinical mechanisms distinguish brain tumour-related SLP disorders from post-stroke deficits. Unlike the abrupt focal destruction within defined arterial territories seen in stroke [42], gliomas infiltrate white matter tracts progressively, permitting extensive pre-lesional neuroplastic reorganisation through perilesional recruitment and contralateral compensation [2,3]. As a result, tumour patients often maintain partial tract functionality and present with subtle, compensated baseline deficits. This structural difference drives a marked domain-specific phenotypic divergence. In language, tumour-related aphasia is dominated by anomic profiles, lexical retrieval delays, and executive-linguistic breakdowns in discourse and pragmatics [22,26] rather than classic stroke syndromes [43]; standard batteries like the WAB or BDAE miss these nuances, creating severe ceiling effects that misclassify impaired patients as intact. Similarly, motor speech deficits in neuro-oncology are highly dynamic, frequently presenting as transient SMA syndrome or articulatory fatigue under cognitive load rather than static post-stroke motor execution failure [43]. Dysphagia also differs, appearing as an insidious, subclinical, or fluctuating impairment driven by post-operative oedema, brainstem compression, radiation, or cognitive decline [39] rather than acute post-stroke pharyngeal failure with high immediate aspiration risk. Furthermore, functional trajectories in tumour patients are non-linear, continuously modulated by oncological confounders such as vasogenic oedema, anti-epileptic drugs, corticosteroids, fatigue, and adjuvant therapies [2,9]. Because traditional stroke tools lack sensitivity to higher-order network disorganisation, executive-linguistic integration, and treatment-modulated fluctuations, they consistently fail in neuro-oncology. While the dynamic organisation previously described explains the predominance of milder baseline phenotypes and high rates of post-operative recovery in tumour patients, the existing literature heavily suffers from reporting bias and cross-sectional design limits. Most studies evaluate patients at arbitrary, non-standardised time points (e.g., immediate post-op vs. 3 months), confounding transient surgical oedema/diaschisis with true permanent network damage. A central conceptual finding of this review is that aphasia, dysarthria, and dysphagia represent manifestations of shared network vulnerability—particularly involving periventricular white matter tracts, the internal capsule, and brainstem nuclei [2,3,15,21].

Despite these anatomical linkages, critical methodological gaps hinder causal inferences: most studies lack integration with quantitative tractography (dTI) and rely on stroke-derived clinical tools that exhibit marked ceiling effects, rendering them insensitive to subtle executive-linguistic or subclinical motor-swallowing breakdowns. This network overlap accounts for the frequent co-occurrence of dysarthria and dysphagia in brainstem and deep frontal lesions, as well as their dissociation in more cortical tumours. Overall, this integrative perspective supports a shift toward an early domain-spanning assessment rather than an isolated, compartmentalised evaluation, and reinforces the need for multidisciplinary management involving neurosurgery, speech-language pathology, neuropsychology, and rehabilitation medicine.

Collectively, these findings support a transition from symptom-based classification toward a connectome-based model of neurogenic communication and swallowing disorders. A comprehensive synthesis of the clinical phenotypes, neuroanatomical correlates, and functional characteristics of aphasia, dysarthria, and dysphagia in brain tumour patients is provided in Table 3.

A further limitation in the current literature relates to the widespread reliance on stroke-derived assessment instruments, as summarised in Table 4, which are inherently limited in their ability to capture the subtle, network-based impairments characteristic of brain tumours. Nevertheless, the results strongly support a shift from localisation-based models toward holistic, network-level frameworks in which language and swallowing functions arise from dynamic connectivity within large-scale networks.

### 4.2. Domain-Specific Clinical Implications

#### 4.2.1. Aphasia

Aphasia remains the most extensively studied SLP disorder in neuro-oncology, yet current findings consistently challenge classical localisationist models. The predominance of anomic aphasia, the low incidence of global aphasia, and the frequent near-complete recovery within months suggest that language functions in slow-growing tumours are supported by redundant and re-organisable networks rather than fixed cortical centres [3,20]. Unlike acute vascular lesions, slow-growing gliomas allow progressive cortical and subcortical reorganisation, leading to partial functional redistribution of language to perilesional and contralateral regions, as demonstrated by functional Magnetic Resonance Imaging (fMRI) and intra-operative mapping studies [2,3]. This neuroplastic adaptation may explain the relatively preserved overt language function frequently observed even in tumours involving classical eloquent regions.

However, impairments in confrontation naming and verbal fluency may persist despite largely preserved global language scores, supporting a network-based and “subclinical” profile of language disruption [22].

Emerging data further indicate that tumours outside classical eloquent regions can still disrupt distributed semantic and executive networks, reinforcing the need for network-sensitive assessment frameworks [23,28]. This challenges the assumption that lesion location alone can predict language outcomes. Evidence from awake brain tumour surgery shows that naming error analysis can effectively reflect underlying semantic and phonological impairments, as error rates correlate directly with domain-specific neuropsychological performance [22]. While group-level results often indicate a general post-surgical decline in language, individual trajectories are more variable, with most patients remaining stable or improving rather than deteriorating [27]. In fact, patients with LGG generally exhibit better cognitive outcomes than those with HGG, likely due to greater neuroplastic capacity. Overall, combining spontaneous speech analysis, naming error profiling, and individualised assessment improves the detection of subtle language deficits that are frequently missed by standard measures. In line with this, recent evidence underscores that post-operative lexical retrieval deficits and spontaneous speech disfluencies act as sensitive markers of underlying network disruption, directly reflecting patients’ subjective communication changes [22]. Importantly, these impairments are also reflected at the subjective level, with a greater number of patients reporting changes in language and communication after surgery. At a more fine-grained level, lexical retrieval impairment has been associated with a higher proportion of false starts, while temporal speech measures such as articulation and speech rates appear sensitive to differences in perceived language change. Overall, these findings suggest that the analysis of spontaneous speech, especially disfluency patterns and temporal speech features, serves as a sensitive marker of lexical retrieval difficulties in individuals with LGG, particularly after surgery [26].

Regarding intra-operative stimulation, studies consistently identified subcortical white matter tracts, most notably the arcuate fasciculus and inferior fronto-occipital fasciculus, as key determinants of persistent language impairment [3,18]. These findings reinforce the central role of white matter structural connectivity, rather than isolated cortical territory, in dictating aphasia severity and recovery. However, a persistent methodological limitation is the reliance on stroke-derived aphasia batteries (e.g., WAB, BDAE) [29,30], which insufficiently capture higher-order linguistic dysfunction. Evidence suggests that tumour patients frequently exhibit subtle impairments in lexical access, discourse organisation, and executive-linguistic integration despite preserved performance on standard measures [9,22,26], likely resulting in a systematic underestimation of clinically relevant deficits. In line with this, recent evidence underscores that post-operative lexical retrieval and spontaneous speech disfluencies act as sensitive markers of underlying network disruption, directly reflecting patients’ subjective communication changes [27].

Conventional prevalence estimates may therefore underestimate early, clinically relevant impairments. A major limitation across studies is the underrepresentation of higher-order language functions, such as discourse, inferencing, and semantic integration. Emerging evidence supports the use of more complex semantic tasks, such as the Pyramids and Palm Tree Test [44] or the revised version of DO 80 [45], which may better predict post-operative outcomes and reflect distributed network integrity. This underscores the need for extended language batteries assessing executive-linguistic functions. Crucially, a major gap in the current literature regarding tumour-related aphasia is the striking scarcity of interventional studies. While substantial research efforts are dedicated to pre-operative mapping, intra-operative assessment, and acute post-operative deficits, the subsequent rehabilitation phase remains significantly under-researched. Consequently, clinical practice almost entirely relies on unvalidated extrapolations from stroke rehabilitation protocols, leaving an ongoing lack of robust evidence concerning the direct efficacy and long-term outcomes of SLP specific to this clinical population. This significant research gap highlights the urgent need for structured, longitudinal clinical trials to establish targeted rehabilitation protocols.

#### 4.2.2. Dysarthria

Compared with aphasia, dysarthria remains markedly under-investigated in neuro-oncology despite its clinical relevance and functional impact. Post-operative dysarthria has been most frequently associated with disruption of corticobulbar pathways, SMA circuits, the ventral premotor cortex, and periventricular white matter [2,3].

SMA syndrome represents a prototypical and often reversible phenotype, characterised by transient speech initiation deficits and mild dysarthria following medial frontal resections, with recovery typically occurring within three months. In contrast, involvement of deep white matter tracts or brainstem pathways is more consistently associated with persistent motor speech impairment.

In awake mapping studies, transient dysarthria or anarthria induced by stimulation of the ventral premotor regions and periventricular fibres further confirms their critical role in motor speech planning and execution [2,3]. These observations support a distributed cortico-subcortical model of motor speech control rather than a purely cortical localisation framework. Critically, the literature is severely limited by two major factors: (1) an over-reliance on subjective perceptual intelligibility scales, which fail to distinguish motor execution deficits from higher-level planning breakdowns, and (2) a profound mismatch between clinician-rated metrics and patient-reported communicative burden.

Perceptual intelligibility ratings lack sensitivity for detecting subtle or subclinical motor speech deficits, yet objective acoustic and kinematic analyses remain largely underutilised despite their potential to quantify articulatory timing and breakdown patterns. [21]. Furthermore, the consistent discrepancy between clinician-rated intelligibility and patient-reported communication difficulty suggests that the functional burden of dysarthria in neuro-oncology is frequently underestimated by standard clinical tools. Without objective acoustic, kinematic, and electromyographic quantification, subtle motor speech deficits remain undetected, while the lack of tumour-specific dysarthria protocols further limits cross-study comparability and hinders the development of targeted rehabilitation strategies.

#### 4.2.3. Dysphagia

Finally, dysphagia is the least studied yet potentially most clinically consequential SLP disorder in brain tumour populations. Reported prevalence estimates vary substantially (15–48%), reflecting heterogeneity in tumour location, timing of assessment, and methodological limitations.

Swallowing impairment reflects the disruption of an integrated cortico-brainstem network involving cortical control regions and the brainstem central pattern generator. In particular, lesions affecting medullary structures or exerting mass effect within the posterior fossa may result in CPD due to impaired coordination of vagal nuclei and upper UES relaxation [36,37,40].

A consistent association has been reported between dysphagia and dysarthria, reflecting the shared involvement of corticobulbar pathways. Evidence from mixed neurological populations indicates high rates of co-occurrence, particularly in conditions affecting bulbar motor systems [21]. In contrast, cerebellar involvement appears less consistently associated with swallowing impairment despite frequent dysarthria, suggesting a partial dissociation of these functions. This dissociation is clinically relevant in brain tumour populations, where posterior fossa lesions may produce motor speech impairment without necessarily compromising swallowing safety. Moreover, cognitive-communicative deficits may further exacerbate swallowing dysfunction by impairing attention, awareness, and executive control during feeding [39]. From a critical standpoint, the primary flaw in existing dysphagia research is the widespread reliance on unvalidated bedside screening tools, which miss up to 50% of silent aspiration events and underestimate clinically significant dysfunction. Instrumental assessments (VFSS, FEES) are inconsistently applied, and tumour-specific swallowing protocols are lacking. Where used, these methods are often extrapolated from stroke populations despite potential differences in underlying pathophysiology. While post-stroke dysphagia typically stems from an acute focal vascular insult affecting specific cortical or subcortical centres [46], brain tumour-related dysphagia involves distinct motor and sensory disruptions unique to swallowing mechanics [15]. Tumours involving the brainstem or posterior fossa directly compress or infiltrate the central pattern generators—specifically the nucleus tractus solitarius and nucleus ambiguus—disrupting the automatic sequencing of the pharyngeal phase [15]. Furthermore, neurosurgical interventions and radiation therapy can disrupt cortical and brainstem swallowing networks, compromising lower cranial nerve integration (CN IX, X, XII). This central disruption impairs laryngeal sensation and motor protection, significantly increasing the risk of silent aspiration [15]. Unlike the vascular insults of stroke, these combined central-to-peripheral insults alter hyolaryngeal elevation, pharyngeal constriction, and UES relaxation in a highly heterogeneous pattern, uniquely constraining neuroplastic adaptation [15].

Consequently, applying stroke-derived frameworks to brain tumour patients overlooks these unique neuromuscular mechanics, creating a critical gap in both assessment and rehabilitation. While stroke care increasingly benefits from standardised structures, such as the International Dysphagia Diet Standardisation Initiative (IDDSI) [47], instrumental assessments and specific treatment protocols are increasingly standardised. The absence of comparable tools in neuro-oncology limits both clinical comparability and translational rehabilitation development. Preliminary evidence suggests that dysphagia is responsive to structured rehabilitation, with outcomes comparable to stroke populations when similar treatment intensity is applied [15]; however, prospective, randomised interventional evidence remains absent.

### 4.3. Limitations

First and foremost, the core methodological limitation of this paper stems from its narrative review design. Unlike systematic or scoping reviews, narrative syntheses do not employ predefined, rigid protocol-driven search protocols or systematic quality appraisal tools (such as the PRISMA guidelines or Risk of Bias frameworks) [48,49]. Consequently, despite our structured approach to search strategy, this design carries an inherent susceptibility to selection, reporting, and publication bias. Beyond these review-level methodological constraints, the current evidence base is constrained by several structural limitations. First, as mentioned before, most studies rely on assessment tools developed for stroke populations, which lack sensitivity to the subtle, fluctuating deficits typical of brain tumours. Second, published cohorts suffer from significant selection bias, predominantly featuring surgical candidates with a high baseline performance (e.g., low-grade gliomas undergoing awake surgery) while systematically under-representing high-grade, deteriorating, or elderly patients. Moreover, there is substantial heterogeneity in tumour characteristics, including the histological subtype, anatomical location, and WHO grade [50], as well as variability in patient age and clinical presentation, all of which limit comparability across studies and the generalisability of findings. The variability in timing of assessment also introduces significant methodological noise, with early evaluations likely overestimating transient deficits and delayed assessments potentially underestimating acute dysfunction.

Additionally, longitudinal data beyond 6–12 months are scarce, limiting our understanding of long-term recovery trajectories, particularly regarding the late neurocognitive and linguistic impact of adjuvant radiochemotherapy.

Ultimately, rehabilitation outcomes remain under-investigated, with a near absence of controlled interventional studies across all SLP domains.

## 5. Conclusions and Future Directions

Despite the methodological limitations, current evidence supports the integration of SLP assessment across the entire brain tumour care pathway. Pre-operative evaluation establishes functional baselines and informs surgical planning, intra-operative mapping enables real-time preservation of critical functional networks, and post-operative rehabilitation is essential for optimising communication, swallowing safety, and quality of life.

Importantly, SLP disorders in primary brain tumours should be conceptualised as manifestations of distributed connectome disruption rather than isolated focal deficits. Across the language, motor speech, and swallowing domains, clinical phenotypes reflect the dynamic interactions between tumour growth, network reorganisation, and treatment-related neurobiological changes.

Within this framework, aphasia most commonly presents as a mild anomic syndrome with substantial recovery potential, dysarthria is often transient and associated with cortico-subcortical or supplementary motor area involvement, and dysphagia remains the most under-recognised yet clinically high-risk manifestation, particularly in tumours affecting the brainstem and deep white matter networks.

SLP disorders frequently co-occur and contribute to a cumulative impact on health-related quality of life, including reduced social participation, psychological distress, and increased caregiver burden.

To bridge the critical evidence gaps identified in this review, future research must transition from descriptive stroke-adapted models toward innovative, technology-driven, and connectome-informed care pathways. Therefore, key priorities and research directions must focus on:

- Development of Tumour-Specific Assessment Batteries: moving away from stroke-derived tools toward validated, standardised batteries designed specifically for neuro-oncology, capable of detecting subtle executive-linguistic breakdowns, discourse alterations, and subclinical aspiration.
- Digital Speech Biomarkers and Automated Speech Recognition: leveraging digital health technologies and automated speech processing algorithms to detect micro-changes in spontaneous speech, lexical retrieval dynamics, and temporal speech parameters, enabling remote, objective, and continuous longitudinal monitoring across the disease trajectory.
- Advanced Acoustic Analysis: integrating high-resolution acoustic and kinematic metrics to complement subjective perceptual intelligibility scales, thereby objectively quantifying subtle articulatory fatigue, motor speech planning breakdowns, and early dysarthric markers.
- Objective Swallowing Metrics and Technology-Assisted Rehabilitation: standardising instrumental dysphagia protocols (VFSS, FEES, High-Resolution Manometry) and developing biofeedback-guided swallowing therapies tailored to brain tumour pathophysiology.
- Controlled Interventional Clinical Trials: executing longitudinal, prospective trials to establish evidence-based SLP rehabilitation protocols specifically evaluated within neuro-oncological cohorts.

Ultimately, advancing neuro-oncological speech-language pathology requires a paradigm shift from reactive management to proactive, connectome-informed precision care. Transforming these research priorities into clinical practice will not only refine our mechanistic understanding of tumour-induced network disruption but will fundamentally enhance functional preservation, long-term autonomy, and holistic quality of life for brain tumour patients and their families.

## Figures and Tables

**Table 1:** Characteristics of included studies investigating speech, language, and swallowing outcomes in brain tumour populations.

Study	Design	Population	Clinical Context and Timing	SLP Domain(s) and Key Focus
Duffau et al. (2002, 2008) [2]	Intra-operative mapping series	Glioma patients (*n* = 250 in 2002; *n* = 65 in 2008); left hemisphere eloquent/non-eloquent regions	Awake craniotomy; intra- and post-operative assessments	Aphasia, dysarthria. Subcortical language and motor speech mapping
Bello and Gallucci (2007) [18]	Intra-operative mapping cohort	Brain tumour patients with lesions in language-eloquent areas	Awake craniotomy; intra-operative and peri-operative monitoring	Aphasia. Intra-operative language testing, cortical/subcortical mapping, and functional preservation
Ilmberger et al. (2008) [19]	Prospective clinical study	Brain tumour patients (*n* = 53) undergoing surgical resection	Pre-operative, post-operative, and short-term follow-ups	Aphasia. Language impairment patterns, executive-linguistic deficits, and early post-surgical course
Davie et al. (2009) [11]	Retrospective cohort	Malignant brain tumour patients (*n* = 95)	Post-operative	Aphasia. Subtype distribution
Satoer et al. (2013) [20]	Prospective cohort	Glioma patients (*n* = 27) (LGG and HGG); left hemisphere eloquent areas	Longitudinal pre-/post-operative follow-ups	Aphasia
Wesling et al. (2013) [15]	Comparative cohort study	Brain tumour patients (*n* = 30) vs. stroke controls (*n* = 30)	Acute and subacute rehabilitation setting	Dysphagia. Biomechanical swallowing characteristics and functional outcomes compared to stroke
Finch and Copland (2014) [9]	Systematic review	Mixed primary and secondary brain tumour populations (12 studies included)	Across studies	Aphasia
Jani and Gore (2014) [21]	Neurological cohort	40 patients with ABI, stroke, tumours	Rehabilitation setting	Dysarthria, dysphagia
Antonsson et al. (2018) [22]	Prospective longitudinal study	LGG patients (pre- and post-surgery)	Pre-op and post-operative follow-ups	Aphasia. Pre-operative language ability and lexical retrieval deficits in LGG
Chang et al. (2018) [23]	Intra-operative study	Glioma patients (*n* = 46); left hemisphere	Awake mapping; intra-/post-operative	Aphasia. Semantic mapping tasks (PPTT)
Fang et al. (2021) [24]	Classification study	Glioma patients (*n* = 164 in prediction cohort); cortical/subcortical regions	Pre-operative	Aphasia risk, location-based prediction model
Collée et al. (2022) [25]	Systematic review	Glioma populations (31 studies included)	Awake surgery; intra-/post-operative	Aphasia. Predictors of language outcome
Antonsson et al. (2024) [26]	Prospective Longitudinal study	Brain tumour patients, LGG patients pre- and post-surgery	Pre-op and post-operative follow-ups	Motor speech/fluency. Disfluency patterns in spontaneous speech before and after surgery
Gasa-Roqué et al. (2024) [27]	Prospective neuropsychological cohort	Brain tumour patients (LGG vs. HGG)	Pre- and post-surgical evaluations	Language and cognitive outcomes and tumour-grade effects on recovery
Klebic et al. (2025) [28]	Clinical cohort	Brain tumour patients (*n* = 120)	Pre-operative	Aphasia. Anomia as an early marker

Abbreviations: LGG = low-grade glioma; HLL = higher-level language; post-op = post-operative; pre-op = pre-operative; 1d = 1 day; 3m = 3 months; PPTT = Pyramids and Palm Trees Test; ABI = acquired brain injury; SLP = speech-language pathology.

**Table 2:** Neuroanatomical correlates of speech, language, and swallowing disorders in brain tumour populations.

SLP Domain	Cortical Regions	Subcortical Tracts	Brainstem Involvement	Functional Profile
Aphasia	Inferior frontal gyrus, superior temporal gyrus, angular gyrus, insula	AF, IFOF, SLF, ScF	Rare; typically indirect (diaschisis)	Anomia, reduced fluency, lexical-semantic and executive-language deficits
Dysarthria	Primary motor cortex (face region), SMA, ventral PMC	Corticobulbar tracts, PVWM pathways, motor-related SLF fibres	Occasional	Mixed spastic–flaccid dysarthria, impaired speech initiation
Dysphagia	Insula, frontal operculum, sensorimotor cortex	Corticobulbar tract, vagal pathways	Frequent; particularly medullary involvement (NTS, DMN)	Oropharyngeal dysphagia, UES dysfunction, aspiration risk

Abbreviations: AF = arcuate fasciculus; DMN = dorsal motor nucleus of the vagus nerve; IFOF = inferior fronto-occipital fasciculus; NTS = nucleus tractus solitarius; PMC = premotor cortex; PVWM = periventricular white matter; ScF = subcallosal fasciculus; SLF = superior longitudinal fasciculus; SMA = supplementary motor area; UES = upper oesophageal sphincter.

**Table 3:** Clinical phenotype summary of SLP disorders.

Disorder	Onset	Phenotype	Course	Key Predictor	Prognosis
Aphasia	Pre-/post-op	Anomic	Transient/mild persistent	AF/IFOF integrity	Favourable (LGG)
Dysarthria	Post-op	SMA syndrome/mixed	Usually transient	SMA, PVWM	Good if cortical
Dysphagia	Post-op/treatment	Oropharyngeal	Variable	Brainstem	Guarded if infratentorial

LGG = low-grade glioma; AF = arcuate fasciculus; IFOF = inferior fronto-occipital fasciculus; SMA = supplementary motor area; PVWM = periventricular white matter.

**Table 4:** Assessment tools used in brain tumour SLP studies.

Domain	Core Tools	Primary Construct	Critical Methodological Limitations and Biases
Aphasia	WAB/WAB-R, BDAE, BNT, DO80	Global language, naming, classification	High ceiling effects; stroke-based norms; insensitive to subtle executive-linguistic, discourse, and subclinical semantic deficits; poor ecological validity
Dysarthria	FDA-2, perceptual ratings, acoustic analysis	Motor speech, intelligibility, speech timing	Subjective perceptual bias; low tumour validation; high inter-rater variability; fails to capture articulatory fatigue and fine motor-planning breakdowns.
Dysphagia	VFSS + PAS, FEES, HRM, bedside assessment	Airway protection, swallowing physiology	Severe underutilization of instrumental tools (VFSS/FEES); reliance on bedside screens with high false-negative rates for silent aspiration; lack of tumour-specific staging protocols.
All domains	PROMs	Patient-reported QoL	Lack of validation in neuro-oncology; confounded by cognitive deficits, anosognosia, mood disorders, and steroid therapy.

WAB = Western Aphasia Battery; WAB-R = revised version; BDAE = Boston Diagnostic Aphasia Examination; BNT = Boston Naming Test; VFSS = videofluoroscopic swallowing study; PAS = Penetration-Aspiration Scale; FEES = fibreoptic endoscopic evaluation of swallowing; HRM = high-resolution manometry; PROMs = patient-reported outcome measures.

## Data Availability

Data supporting the findings of this narrative review were extracted from published studies and are available within the cited references. No new primary data were generated in this study.

## Abbreviations

The following abbreviations are used in this manuscript:

SLP	Speech and Language Pathology
HRQoL	Health-Related Quality of Life
LGG	Low-Grade Glioma
HGG	High-Grade Glioma
HLL	High-Level Language
ABI	Acquired Brain Injury
AF	Arcuate Fasciculus
DMN	Dorsal Motor Nucleus of the vagus nerve
IFOF	Inferior-fronto-occipital fasciculus
NTS	Nucleus Tractus Solitarius
PMC	Premotor Cortex
PVWM	Periventricular White Matter
ScF	Subcallosal fasciculus
SLF	Superior Longitudinal Fasciculus
SMA	Supplementary Motor Area
UES	Upper Oesophageal Sphincter
VFSS	Videofluoroscopic Swallowing Study
FEES	Fibreoptic Endoscopic Evaluation of Swallowing
CSE	Clinical Swallowing Evaluation
PEG	Percutaneous Endoscopic Gastrostomy
RIG	Radiologically Inserted Gastrostomy
HRM	High-Resolution Manometry
PROMs	Patient-Reported Outcomes Measures
fMRI	Functional Magnetic Resonance Imaging
CPD	Cricopharyngeal Dysfunction
